# Body mass index and quality of life among outpatients with schizophrenia in Japan

**DOI:** 10.1186/1471-244X-13-108

**Published:** 2013-04-09

**Authors:** Norio Sugawara, Norio Yasui-Furukori, Yasushi Sato, Manabu Saito, Hanako Furukori, Taku Nakagami, Shuhei Kudo, Sunao Kaneko

**Affiliations:** 1 Department of Neuropsychiatry, Hirosaki University School of Medicine, Hirosaki, Japan; 2 Department of Psychiatry, Hirosaki-Aiseikai Hospital, Hirosaki, Japan; 3 Department of Psychiatry, Kuroishi-Akebono Hospital, Kuroishi, Japan; 4 Department of Psychiatry, Odate Municipal General Hospital, Odate, Japan

**Keywords:** Body mass index, Quality of life, Schizophrenia, Japan

## Abstract

**Background:**

Obesity is becoming more prevalent and thus growing as a public health concern in patients with schizophrenia. This investigation evaluated the relationship between body weight and the self-reported quality of life (QOL) of Japanese patients with schizophrenia.

**Methods:**

We recruited outpatients (n=225) aged 42.5 ± 12.8 (mean ± SD) years with a DSM-IV diagnosis of schizophrenia who were admitted to psychiatric hospitals. This study used a cross-sectional design. The assessments included an interview to obtain sociodemographic data, the second version of the Short Form Health Survey (SF-36v2), the 10-item version of the Drug Attitude Inventory (DAI-10), the Clinical Global Impression-Severity (CGI-S) and height and weight measurements. SF-36v2 subscores were examined for differences based on the following body mass index (BMI) categories: healthy weight (BMI < 24.9), overweight (BMI 25–29.9) and obese (BMI > 30). A multiple regression analysis was employed to assess the relationship between these BMI categories and QOL outcomes.

**Results:**

The overall prevalence of obesity in our sample was 16.4%. A multiple regression model revealed that age, gender, DAI-10 scores, CGI-S scores, social functioning, role emotional functioning, mental health, and Mental Composite Summary (MCS) score were significantly and positively associated with overweight status. Physical functioning, general health, role emotional functioning, mental health, and a physical composite summary (PCS) score were significantly and negatively associated with obesity.

**Conclusions:**

The burden of obesity is both a physical and a mental problem. An obesity intervention program for patients with schizophrenia may improve health-related QOL in patients with schizophrenia.

## Background

Obesity is a growing public health concern, as is becoming more prevalent among patients with schizophrenia compared with the general population [1-3]. Previous studies have shown that being overweight is a major risk factor for metabolic syndrome, cardiovascular diseases, and premature death. Furthermore, this risk is nearly twice that of the general population among patients with schizophrenia [4-6]. In addition, obesity among patients with schizophrenia is associated with high medication costs [7], low self-esteem, poor psychosocial adaptation [8], non-compliance with an antipsychotic medication regime [9] and reduced quality of life (QOL) [10].

QOL can be defined as the impact of illness and condition on the physical and mental functioning from the point of view of the patient. Patients with schizophrenia have low QOL [11]. Previous studies of Western populations have shown that the QOL of patients with schizophrenia further decreased with obesity [10,12-14]. However, we are not aware any study concerning this issue among Asian populations, who have a different obesity prevalence and lifestyle from Western populations [15-17]. QOL can be used to assess how patients feel and function in their everyday life with regard to a treatment, and a good QOL may improve the measurement of treatment efficacy. Directly treating QOL concerns can both improve a patient’s QOL and attenuate symptoms of the disorder [18,19]; thus, understanding the association between obesity and QOL would be useful.

This investigation evaluated the relationship between body weight and the self-reported QOL of Japanese patients with schizophrenia. To our knowledge, this study is the first of this nature conducted with an Asian population.

## Method

### Participants

This study was conducted between June 2011 and August 2011. The participants included 225 outpatients (106 males and 119 females) from four psychiatric hospitals in Japan who were diagnosed with either schizophrenia or schizoaffective disorder according to the Diagnostic and Statistical Manual of Mental Disorders, Fourth Edition (DSM-IV) diagnostic criteria. Patients’ diagnoses established by experienced psychiatrists responsible for their treatment were recorded from their medical charts. The Ethics Committee of the Hirosaki University School of Medicine approved the data collection for this study, and all participants provided written informed consent before volunteering.

### Procedure

Participant demographic data (age and sex) were obtained from their medical records. Participant height and weight were measured, and body mass index (BMI) was calculated for each participant. Participants were classified as having a normal weight if their BMI was below 25, overweight if their BMI was between 25 and 29.9, and obese if their BMI was 30 or above. The Clinical Global Impression-Severity (CGI-S) score was used to measure symptom severity. The CGI-S asks the clinician one question: “Considering your total clinical experience with this particular population, how mentally ill is the patient at this time?” This question is rated from 1 (normal, not at all ill) to 7 (among the most extremely ill patients). The Drug Attitude Inventory (DAI) is a self-applied scale that measures subjective responses to medication. This instrument reveals whether the patient is satisfied with their treatment and evaluates their understanding of how the treatment is affecting them. The reduced version of the DAI (DAI-10) has ten highly specific items concerning the participant’s subjective experience. These items are based on the recorded and transcribed accounts of the patients, and the response options are true or false. These items were selected for their ability to discriminate between grades of medication adherence in a way that could be analyzed.

The Short Form Health Survey, Version 2 (SF-36v2) was used to assess participants’ health-related QOL. The SF-36v2 is a standardized, 36-item, self-administered questionnaire that was translated, adapted, and validated for use in Japan [20,21]. This questionnaire assesses eight QOL domains of health status. The domains concerning physical health consist of physical functioning, role physical functioning, body pain, and general health. The domains concerning mental health consist of vitality, social functioning, role emotional functioning, and mental health. For each QOL domain, a score ranging from 0 to 100 is calculated, and higher scores indicate more positive perceptions of health-related QOL. In addition, the scores from all eight domains are combined to create more comprehensive indicators of physical and mental health: the Physical Composite Summary (PCS) and the Mental Composite Summary (MCS). The PCS and MCS are standardized (Japanese mean = 50, standard deviation = 10) to compare with the general population or the results of other studies.

### Statistical analyses

Descriptive analyses were performed on the demographic and clinical variables. An analysis of variance (ANOVA) and the Tukey post-hoc test were performed to compare the primary continuous demographic and clinical characteristics between groups, and a chi-square test was performed to analyze categorical variables. Data are presented as the mean ± SD. A multiple linear regression was employed to analyze the effects of obesity on the SF-36v2 continuous variables. Regression analyses were conducted to adjust for confounding factors (age, gender, DAI-10, and CGI-S). A value of p<0.05 was considered significant. The data were analyzed using PASW Statistics software for Windows, version 18.0.0.

## Results

### Demographic characteristics

Table 1 presents the participants’ characteristics. The obese group, but not the overweight group, showed lower physical functioning score compared with the healthy weight group. General health and role emotional functioning scores were higher for the overweight group compared with the obese group. The overweight group, but not the obese group, had higher MCS scores compared with the healthy weight group. No differences were observed with regard to the other characteristics.

**Table 1:** Characteristics by BMI group

		**Body mass index**			
	**Total**	**Healthy weight**	**Overweight**	**Obesity**	**ANOVA**
		BMI<25	25≦BMI<30	BMI≧30	p value
n	225	123	65	37	
Age	42.5±12.8	42.0±13.6	44.6±11.4	40.4±12.2	0.234
Gender	Male 106	Male 54	Male 36	Male 16	0.284
	Female 119	Female 69	Female 29	Female 21	
Height	163.7±8.9	163.3±8.8	165.0±9.1	162.9±9.4	0.383
Weight	68.0±15.7	58.2±9.2	73.9±9.3	90.5±13.5	<0.001 ^a^
Clinical Global Impression Severity scale	3.1±1.0	3.1±1.0	3.2±1.0	3.2±0.9	0.521
Drug Attitude Inventory-10	6.9±2.3	6.9±2.2	6.7±2.3	7.3±2.4	0.496
Short Form Health Survey Version 2					
Physical functioning	44.3±12.9	46.1±11.0	44.1±13.8	38.7±15.8	<0.01 ^b^
Role physical functioning	42.0±13.5	42.5±13.9	42.6±12.2	39.4±14.4	0.431
Body pain	49.2±11.1	48.6±10.8	50.8±11.5	48.5±11.5	0.384
General health	45.6±10.5	45.5±10.0	47.6±10.2	42.0±11.7	<0.05 ^c^
Vitality	45.2±11.4	44.7±11.8	47.3±11.1	43.0±10.6	0.155
Social functioning	42.0±13.4	40.8±14.0	44.9±11.6	40.6±14.2	0.112
Role emotional functioning	40.2±14.2	39.5±14.3	43.8±12.0	36.4±16.4	<0.05 ^c^
Mental health	43.7±11.3	43.0±11.1	46.3±11.2	41.3±11.3	0.057
Physical composite score	42.2±13.0	43.1±12.5	43.0±12.1	38.1±15.6	0.108
Mental composite score	46.0±11.0	44.8±11.0	48.9±10.7	44.7±10.4	<0.05 ^d^

Data are presented as the means ± SD.

a Indicates a significant difference (P < 0.05) between the healthy weight group versus the overweight group, the healthy weight group versus the obesity group and the overweight group versus the obesity group.

b Indicates a significant difference (P < 0.05) between the healthy weight group and the obesity group.

c Indicates a significant difference (P < 0.05) between the overweight group and the obesity group.

d Indicates a significant difference (P < 0.05) between the healthy weight group and the overweight group.

ANOVA = analysis of variance.

### Factors that influenced the SF-36

Table 2 shows the multiple regression results for the SF-36 scores. The physical functioning, role emotional functioning, and mental health domains and the PCS and MCS scores were significantly associated with age. Role physical functioning and PCS score were significantly and positively associated with gender (being male). All eight domains of the SF-36 and the PCS and MCS scores were significantly associated with the CGI-S scores. Role physical functioning, body pain, general health, vitality, social functioning, mental health, and the MCS score were significantly associated with the DAI-10 scores. Social functioning, role emotional functioning, mental health, and the MCS score were significantly and positively associated with an overweight status. Physical functioning, general health, role emotional functioning, mental health, and PCS score were significantly and negatively associated with obesity.

**Table 2:** Factors that influenced the Short Form 36 (SF-36) scores

		**Multiple regression statistics**				
	**Independent variables**	**B**	**SE**	**β**	**t value**	**p value**
Physical functioning	Age	−0.493	0.087	−0.352	−5.681	<0.001
	Gender **(being male)**	4.019	2.180	0.112	1.844	0.067
	CGI-S	−3.931	1.100	−0.218	−3.574	<0.001
	DAI-10	0.631	0.486	0.080	1.298	0.196
	Overweight	−1.275	2.513	−0.035	−0.507	0.612
	Obesity	−9.400	3.379	−0.195	−2.782	<0.01
Role physical functioning	Age	−0.138	0.129	−0.070	−1.073	0.285
	Gender **(being male)**	7.725	3.235	0.152	2.388	<0.05
	CGI-S	−7.770	1.632	−0.304	−4.760	<0.001
	DAI-10	1.799	0.721	0.162	2.495	<0.05
	Overweight	1.299	3.729	0.025	0.348	0.728
	Obesity	−6.875	5.014	−0.100	−1.371	0.172
Body pain	Age	−0.154	0.127	−0.079	−1.206	0.229
	Gender **(being male)**	4.775	3.198	0.096	1.493	0.137
	CGI-S	−7.642	1.614	−0.304	−4.735	<0.001
	DAI-10	1.743	0.713	0.160	2.446	<0.05
	Overweight	6.441	3.687	0.129	1.747	0.082
	Obesity	−6.340	4.957	−0.094	−1.279	0.202
General health	Age	0.010	0.099	0.007	0.101	0.919
	Gender **(being male)**	1.648	2.486	0.042	0.663	0.508
	CGI-S	−5.579	1.2544	−0.282	−4.447	<0.001
	DAI-10	1.885	0.554	0.219	3.403	<0.01
	Overweight	4.915	2.866	0.125	1.715	0.088
	Obesity	−11.389	3.854	−0.215	−2.955	<0.01
Vitality	Age	0.134	0.115	0.077	1.167	0.245
	Gender **(being male)**	2.645	2.892	0.059	0.915	0.361
	CGI-S	−4.662	1.459	−0.208	−3.195	<0.01
	DAI-10	1.684	0.644	0.173	2.614	<0.05
	Overweight	5.348	3.333	0.120	1.604	0.110
	Obesity	−8.476	4.482	−0.141	−1.891	0.060
Social functioning	Age	0.239	0.131	0.117	1.822	0.070
	Gender **(being male)**	1.076	3.298	0.021	0.326	0.745
	CGI-S	−7.187	1.664	−0.274	−4.319	<0.001
	DAI-10	2.213	0.735	0.194	3.012	<0.01
	Overweight	8.733	3.801	0.167	2.297	<0.05
	Obesity	−8.503	5.111	−0.121	−1.664	0.098
Role emotional	Age	0.327	0.143	0.147	2.294	<0.01
functioning	Gender **(being male)**	6.848	3.585	0.120	1.910	0.057
	CGI-S	−8.015	1.809	−0.280	−4.431	<0.001
	DAI-10	1.192	0.799	0.096	1.492	0.137
	Overweight	8.428	4.132	0.148	2.040	<0.05
	Obesity	−13.373	5.556	−0.174	−2.407	<0.05
Mental health	Age	0.277	0.105	0.169	2.647	<0.01
	Gender **(being male)**	2.443	2.631	0.058	0.929	0.354
	CGI-S	−4.804	1.327	−0.228	−3.620	<0.001
	DAI-10	1.912	0.586	0.209	3.262	<0.01
	Overweight	6.380	3.032	0.152	2.104	<0.05
	Obesity	−9.008	4.077	−0.160	−2.209	<0.05
Physical composite score	Age	−0.186	0.065	−0.182	−2.873	<0.01
	Gender **(being male)**	4.153	1.623	0.159	2.558	<0.05
	CGI-S	−4.200	0.819	−0.320	−5.127	<0.001
	DAI-10	0.624	0.362	0.110	1.726	0.086
	Overweight	0.660	1.871	0.025	0.353	0.725
	Obesity	−5.544	2.516	−0.158	−2.204	<0.05
Mental composite score	Age	0.183	0.054	0.213	3.364	<0.01
	Gender **(being male)**	0.035	1.364	0.002	0.026	0.979
	CGI-S	−2.211	0.688	−0.200	−3.212	<0.01
	DAI-10	1.024	0.304	0.214	3.369	<0.01
	Overweight	4.163	1.572	0.189	2.648	<0.01
	Obesity	−4.067	2.114	−0.138	−1.924	0.056

## Discussion

The present study is the first to examine the association between obesity and the QOL of patients diagnosed with schizophrenia in an Asian population. In this sample, 16.4% of participants with schizophrenia were obese. After adjusting for confounds, three domains of mental health and the MCS score were significantly and positively associated with an overweight status. In addition, obesity was significantly and negatively associated with two domains of physical health, two domains of mental health, and the PCS score in the same model.

Previous studies have found a relationship between obesity and QOL among patients with schizophrenia in Western populations [10,12,14,22]. Allison and colleagues investigated the relationship between QOL and weight gain among 286 patients with schizophrenia. After adjusting for confounds, they found that weight gain was significantly associated with a poorer overall QOL score according to a 16-item scale. Another study from the US used the SF-36 and found an association between obesity and some QOL items among 143 patients with schizophrenia. Worse physical functioning, general health, role emotional functioning, and a lower PCS score were observed among obese participants. Faulkner and colleagues reported that a PCS score of SF-12 was associated with BMI and waist circumference among 90 patients with schizophrenia. Furthermore, Kolotkin and colleagues studied 111 patients with schizophrenia and 100 patients with bipolar disorder and found that obese patients had poorer vitality, social functioning, role emotional functioning, and mental health and lower MCS scores than those patients who were not obese. The contradictory results concerning the overweight group in our study may be due to cultural or ethnic differences. Asian participants who were overweight may have fewer negative attitudes regarding their weight than Western participants of a similar weight [23,24]. Another explanation is that the prevalence of obesity in our study was lower than that of the studies of Western populations. In addition, some studies have compared each QOL domain among patients with schizophrenia using a mixed subject pool of patients who were either overweight or obese.

Our findings have implications for clinicians who treat patients with schizophrenia. First, obesity adds to the burden of schizophrenia not only via physical health risks but also reduced health-related QOL. The cause of obesity among patients with schizophrenia has not been determined completely. However, patients with schizophrenia are at risk for developing obesity due to poor dietary habits, lower resting energy expenditures, a lack of exercise, and limited activity due to their negative symptoms [25,26]. Previous studies have shown that non-pharmacological interventions can reduce body weight [22,27]. Effective treatments are necessary, and these range from nutritional interventions to cognitive behavioral therapy. Second, a pervasive impairment in QOL among patients with schizophrenia may cause poor adherence or even premature discontinuation of treatment because of weight gain. A previous study of patients with schizophrenia showed that both BMI and subjective distress from weight gain predicted noncompliance with medications even after adjusting for other possible confounds [9]. Obese patients are also more than twice as likely as those patients with normal BMIs to report noncompliance with medication.

The present study is limited by its cross-sectional design; thus, we cannot determine a causal relationship between obesity and QOL among patients with schizophrenia. A follow-up survey must be conducted. The second limitation of this study was the patient recruitment, which was restricted to outpatients admitted to the hospital for a review of their health problems. These individuals may not represent all patients with schizophrenia (e.g., children, adolescents, or non-medicated patients). Third, not all possible parameters were included in this study, such as socio-economic status, dietary habits, physical activity levels, the duration of illness and treatment, schizophrenic symptoms and medications. Above mentioned parameters are known to be independently associated with health related QOL or lifestyle, also in patients with schizophrenia [28,29]. In particular, the presence of antipsychotic medications may be an important factor. The use of first- versus second-generation antipsychotics may also affect the results. A stratified analysis by medication is needed in a future study. Fourth, data on validity of the SF-36v2 were lacking in Japanese patients with schizophrenia. Interpretation of our results was hampered by lacking data of the validation study for SF-36 v2.

## Conclusions

Obesity has a significant and negative impact on the QOL of patients with schizophrenia regardless of symptom severity and their attitudes toward antipsychotics. Previous studies suggest that long-term programs that incorporate nutrition, exercise, and behavioral interventions can prevent weight gain among patients with schizophrenia. An intervention program aimed at reducing obesity has the potential to improve patient health-related QOL among patients with schizophrenia.

## Competing interests

The authors declare that they have no competing interests.

## Authors’ contributions

NS conceived the study, designed the study, conducted the statistical analysis, interpreted the data and wrote the initial draft of the manuscript. SKK had full access to all of the data in the study and takes responsibility for the integrity of the data and the accuracy of the data analysis. NYF and SKD contributed to study design and assisted in drafting the manuscript. YS and HF completed initial survey construction, recruitment of participants. MS and TN participated in the data collection, and the interpretation of the results. All authors have approved the manuscript.

## Pre-publication history

The pre-publication history for this paper can be accessed here:

http://www.biomedcentral.com/1471-244X/13/108/prepub
